# Cinematic narrative transportation and prosocial tendencies in college students: a moderated mediation model

**DOI:** 10.3389/fpsyt.2026.1976033

**Published:** 2026-10-21

**Authors:** Yifei Tang, Man Chen, Yujun Deng, Cheng Wei, Yaoyao Zhou, Lingjuan Wang, Yisong Yang

**Affiliations:** 1 School of Performing Arts, Sichuan University of Media and Communications, Chengdu, China; 2 College of Education, Chengdu College of Arts and Sciences, Chengdu, China; 3 SUMC-NYFA Joint School, Sichuan University of Media and Communications, Chengdu, China

**Keywords:** character identification, film psychology, moderated mediation, moral identity, narrative transportation, prosocial tendencies

## Abstract

**Background:**

Narrative-based entertainment has been associated with audience members’ values and behavioral tendencies, yet the mechanisms linking cinematic narrative experiences to prosocial tendencies remain unclear. Drawing on narrative transportation theory and character identification theory, this study examined whether character identification mediates the association between cinematic narrative transportation and prosocial tendencies among college students and whether moral identity moderates the second stage of this indirect pathway.

**Methods:**

A cross-sectional film-viewing survey was conducted with 1054 Chinese undergraduates (*M* = 20.64 ± 1.62 years; 60.06% female). After viewing *Dying to Survive*, participants completed the narrative engagement scale, character identification scale, moral identity scale and prosocial behavior measure(used to assess prosocial tendencies). PROCESS Models 4 and 14, with 5,000 bootstrap samples, were used to test the mediation and moderated mediation models.

**Results:**

Narrative transportation was positively correlated with prosocial tendencies (*r* = 0.67, *p* < 0.01). Character identification partially explained the association (indirect effect = 0.012, Boot 95% CI excluding zero;2.72% of the total effect). Moral identity moderated the character identification–prosocial tendencies pathway (*b* = 0.106, *p* = 0.009): the association was significant at moderate and high levels of moral identity (*b* = 0.090 and 0.139, respectively, *p* < 0.001), but not at a low level (*b* = 0.013, *p* = 0.691). The conditional indirect effects followed the same pattern (moderate: indirect effect = 0.014, 95% Boot CI [0.007, 0.023]; high: indirect effect = 0.022, 95% Boot CI [0.011, 0.034]; index of moderated mediation = 0.017, 95% Boot CI [0.005, 0.031]).

**Conclusion:**

Cinematic narrative transportation was associated with college students’ prosocial tendencies partly through character identification, with moral identity serving as a moderating factor in this process. Film-based aesthetic education featuring moral growth arcs may be related to prosocial tendencies, particularly among students with higher moral identity.

## 1 Introduction

Film serves not only as a source of entertainment but also as a potent narrative medium. The ways, structures, and techniques through which films tell stories constitute their narratives. Narrative is a core component of cinematic art, involving the organization, presentation and communication of story content to audiences (1, 2). Do audiences truly receive the content conveyed by films, and is that content related to their behavior? This question has attracted considerable scholarly interest. Studies have shown that elements of film narratives are associated with audience psychology; for instance, narrative structure has been shown to be associated with emotional resonance, cognition, thought, and psychological satisfaction (3). Relevant literature also indicates that narrative pacing and tension can be associated with audience attention (4), emotional engagement and release, and emotional elevation (5, 6), as well as with audience curiosity (7).

Film consumption constitutes a substantial part of young people’s everyday media life: according to the China Film Administration, China’s total box office reached 42.50 billion yuan in 2024, with 1.01 billion urban cinema admissions nationwide (8). At the same time, the psychological well-being and social functioning of college students have become a pressing public concern: a national survey of nearly 80,000 Chinese college students found that approximately 21.48% were at risk of depression and 45.28% at risk of anxiety (9), and prosocial tendencies have been linked to interpersonal adjustment and mental health in this population (10, 11). Clarifying whether and how cinematic narrative experiences are associated with prosocial tendencies is therefore of direct practical urgency for the design of film-based aesthetic education in universities.

Furthermore, different narrative perspectives are associated with psychological factors at different levels: first-person narrative is associated with greater audience empathy, third-person narrative provides a more comprehensive cognitive framework, and omniscient narrative is linked to a greater sense of control (12, 13). Building on this, researchers are increasingly concerned with how film narrative concretely relates to the public’s values and their behavioral tendencies in reality. The importance of prosocial tendencies in today’s world is self-evident, as such voluntary actions that benefit others or society are relevant to individual psychology, group behavior, and social security (10, 11). Does film narrative relate to prosocial tendencies in the real world? The study by Chen and Lin (14) empirically examined the persuasiveness of *Fly Away Home* (directed by Carroll, 1996) (15) regarding environmental attitudes and prosocial tendencies. Their findings indicated that altruistic behavior can be positively associated with narrative entertainment. Murphy et al. (16) examined how narrative transportation relates to attitude and behavior change, including prosocial tendencies, through character involvement. Zhao and Raney (17) also verified the role of meaningful films on helping behavior. In other words, film narrative may play a role in prosocial tendencies in the public’s reality.

How does film narrative relate to prosocial tendencies in mass reality? Researchers have also conducted studies on this. The narrative transportation theory proposed by Green and Brock (18) suggests that narrative transportation is a distinctive psychological process of “entering the story world.” It involves the integration of attention, emotion, and imagery, whereby an individual’s mental systems and capacities are focused on the narrative events (19). When audiences are highly immersed in the narrative, a state of detachment from reality may occur, reducing critical scrutiny of the narrative content. This state of narrative transportation can weaken the anchoring of audiences’ preexisting attitudes, thereby making them more receptive to and more likely to internalize the moral messages within the narrative. This theory proposes a theoretical pathway: narrative transportation engages the audience’s attention focused on the story world, which is connected to empathy with the story characters, temporarily reconstructs the audience’s own values, and finally is linked to changes in prosocial tendencies. Cohen (20) account of character identification can also help explain this process. Cohen argued that character identification is the motivation for audience members to psychologically take on a character’s perspective and experience the character’s emotions. The self-expansion model suggests that narrative transportation can expand the boundaries of the self, incorporate “others” into the self-concept, thereby corresponding to changes in self-concept and a need for behavioral consistency. Batson (21) empathy-altruism hypothesis indicates that narrative transportation can be associated with emotional resonance, which is associated with empathic concern for characters or groups, thereby being associated with altruistic motivation and ultimately with changes in prosocial tendencies. Synthesizing the above research, we find that narrative transportation has been consistently associated with people’s behavior, and this association is accounted for by factors such as emotional resonance, character identification, and self-concept updating. Therefore, we propose that mediating factors may exist in the association between film narrative transportation and prosocial tendencies. Based on this, the hypotheses of this paper are:

H1: Narrative transportation is positively associated with character identification.

H2: Character identification is positively associated with prosocial tendencies.

H3: Character identification indirectly accounts for the association between narrative transportation and prosocial tendencies. Specifically, narrative transportation is expected to have a positive indirect association with prosocial tendencies through character identification.

However, the existing literature leaves two critical theoretical gaps unaddressed. First, while narrative transportation research has established that immersion is associated with attitude change (18), the specific mechanism through which such immersion is associated with prosocial tendencies—particularly via the affective process of character identification—remains underexplored in the context of full-length cinematic narratives. Most prior studies have focused on short, custom-designed narratives, leaving it unclear whether the same mediating pathway operates when audiences view full-length commercial films in everyday classroom settings. Second, and more importantly, prior work has largely treated narrative effects as universal, implicitly assuming that all transported viewers are equally likely to link character identification with prosocial tendencies. This assumption overlooks the potential role of viewers’ stable self-concept—specifically, moral identity—as a boundary condition that may amplify or attenuate the association between identification and behavior. Although moral identity has been studied in traditional moral decision-making contexts (22), its function within narrative persuasion processes has received limited empirical attention. Therefore, the present study aims to address these gaps by specifying and examining a moderated mediation model specifying how cinematic narrative transportation is associated with prosocial tendencies through character identification and for whom this association may be stronger or weaker depending on moral identity.

The positioning of moral identity as a moderator at the second stage in the pathway from character identification to prosocial tendencies is grounded in self-consistency theory. (23, 24) and the self-expansion model (25, 26). We argue that the moderating role of moral identity is theoretically most plausible at this specific stage for the following reasons. During narrative immersion, character identification blurs the self-other boundary, making the character’s traits and goals momentarily accessible as part of the viewer’s self-concept. However, the translation of this temporarily expanded self-concept into stable prosocial tendencies hinges on whether the character’s moral attributes (e.g., compassion, altruism) are congruent with the viewer’s chronic moral self-schema. When moral identity is high, moral traits are central to the self-concept; thus, incorporating a character’s altruistic actions reinforces the self-concept and is associated with intrinsic pressure to maintain consistency, being associated with the shift from identification to action. Conversely, when moral identity is low, moral traits are peripheral to the self-concept; identifying with a moral character may feel non-self-relevant, thereby being associated with weaker motivational force to act prosocially. Notably, this reasoning does not predict moderation at the first stage in the pathway from narrative transportation to character identification, because the formation of identification—which depends on narrative quality, perspective-taking, and emotional resonance—is primarily driven by situational narrative cues and individual differences in empathy, rather than by moral self-relevance. Likewise, moderation on the direct path from narrative transportation to prosocial tendencies is less theoretically specified, as this direct association may be explained by other mechanisms (e.g., general arousal or mood). Therefore, we position moral identity specifically as a second-stage moderator, hypothesizing that it conditions the strength of the indirect pathway by moderating the link between character identification and prosocial tendencies. Based on this, we propose:

H4: Moral identity moderates the second stage of the indirect pathway, such that the positive association between character identification and prosocial tendencies, and the corresponding indirect association of narrative transportation on prosocial tendencies via character identification, is stronger for individuals with higher rather than lower levels of moral identity.

This hypothesis concerns statistical moderation rather than experimental manipulation. Based on the fundamental logical relationships of the four hypotheses, this paper constructs the following research model (**Figure 1**).

**Figure 1:**
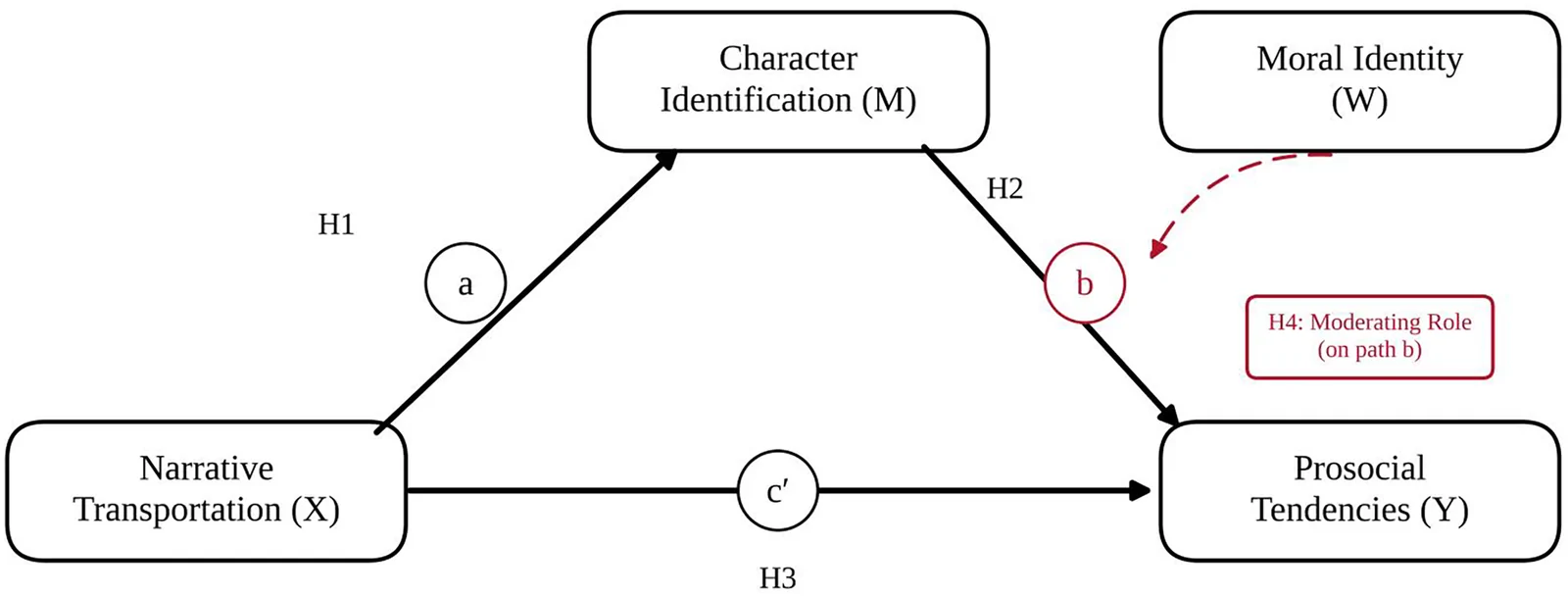
Theoretical model. PROCESS Model 14 (27). X, Narrative Transportation; M, Character Identification; Y, Prosocial Tendencies; W, Moral Identity. H1, Narrative Transportation – Character Identification; H2, Character Identification – Prosocial Tendencies; H3, character identification indirectly accounts for the association between narrative transportation and prosocial tendencies; H4, moral identity moderates the association between character identification and prosocial tendencies.

## 2 Materials and methods

This study used a cross-sectional survey design. Participants were not randomly assigned to conditions, and no control or comparison group was included. All participants viewed the same full-length film and then completed the questionnaires. Therefore, the design does not permit strong causal inference.

Participants. The participants in this study were 1054 undergraduates from Chengdu College of Arts and Sciences (421 men, 633 women), aged 18–23 years (*M* = 20.64, *SD* = 1.62). All participants had normal vision or corrected-to-normal vision, not color-blind or color-deficient and had no background in psychology.

To safeguard participants’ right to informed consent in compliance with ethical guidelines, all participants were fully informed of the basic purpose and procedures of the study before their participation.

### 2.1 Materials

This study investigates the association between narrative transportation and prosocial tendencies, with a focus on the mediating role of character identification and the moderating role of moral identity. Therefore, researchers fully considered the characteristics of these variables when selecting film materials. The film shown to participants was the Chinese film *Dying to Survive*. This realist film was directed by Wen Muye and starring Xu Zheng. It was adapted from the true story of Lu Yong, a patient with chronic myeloid leukemia. The film tells the story of Cheng Yong, the owner of an Indian “magic oil” shop, who smuggles inexpensive generic drugs (Gleevec) from India to treat leukemia patients, profoundly exploring the complex relationship between the medical system, social reality, and human morality. As the character Cheng Yong exhibits a morally contradictory trajectory, it is relatively suitable for measuring participants’ moral identity. This film was selected to reduce potential bias associated with a single, unambiguous moral perspective.

### 2.2 Measures

This study used four scales.

The narrative engagement scale, developed by Rick Busselle and Helena Bilandzic in 2009, is based on the theory of narrative transportation. It conceptualizes transportation as a multidimensional state of narrative experience, is highly correlated with Green and Brock’s transportation scale (*r* = 0.73-0.86), and includes four dimensions: narrative understanding, attentional focus, emotional engagement, and narrative presence, and comprises a total of 12 items. A 7-point Likert scale was used (1 = strongly disagree, 7 = strongly agree). Some items required reverse scoring. Higher scores indicate a greater degree of narrative transportation. The total scale had a Cronbach’s *α* = 0.80. Although the narrative engagement scale was originally developed in Western contexts, it has been applied across diverse cultural settings, including Asian samples, and its psychometric properties have been empirically supported in cross-cultural studies (28, 29). In the present study, the total scale had a Cronbach’s *α* = 0.76; the coefficients for narrative understanding were 0.94, attentional focus at 0.83, narrative presence at 0.77, and emotional engagement at 0.74.

#### 2.2.1 Character identification scale

Developed by Juan-José Igartua in 2010 (30) and validated in collaboration with I. Barrios in 2012. The scale is based on social identity theory and narrative transportation theory, measuring the degree of individual identification with specific media characters, emphasizing emotional involvement and overlap between the self and the character’s role, comprising 11 items. The scale is a 5-point Likert scale (1 = not at all, 5 = very strongly), with an average score calculated from the 11 items; higher scores indicate stronger character identification. Internal consistency was Cronbach’s *α* = 0.86-0.91 (slight variations across different characters). The character identification scale has been validated across multiple cultural contexts in prior research (31). In this Chinese undergraduate sample, the scale demonstrated acceptable internal consistency (Cronbach’s α = 0.70), which is consistent with previous applications of the scale in non-Western settings.

#### 2.2.2 Moral identity scale

Developed by Karl Aquino and Americus Reed II in 2002, this scale is based on social identity theory and self-concept theory, measuring the centrality of moral traits in an individual’s self-concept. The moral identity network is measured by presenting nine moral trait words (caring, compassionate, fair, friendly, generous, helpful, hardworking, honest, kind). The scale is divided into internalization and symbolization dimensions, with a total of 11 items. A 5-point Likert scale was used (1 = strongly disagree, 5 = strongly agree). Each dimension is scored separately, and a total score can also be calculated: the sum of the two dimensions or the average of all 11 items. Previous research has reported good reliability for the internalization dimension: Cronbach’s *α* = 0.83-0.90, symbolization dimension: Cronbach’s *α* = 0.66-0.81, total scale Cronbach’s *α* = 0.78-0.82. The moral identity scale has been translated into Chinese and its construct, convergent, and discriminant validity have been empirically supported in Chinese samples (32). In the present study, the scale demonstrated good internal consistency (internalization dimension: *α* = 0.78; symbolization dimension: *α* = 0.84; total scale: *α* = 0.85). It should be noted that moral identity was measured after the film-viewing session. Theoretically, moral identity is conceptualized as a relatively stable, dispositional self-schema (22). However, because it was assessed immediately after the film, we cannot rule out the possibility that the film-viewing experience temporarily activated or influenced participants’ reports of moral identity. Therefore, moral identity should be interpreted as a relatively stable individual difference that may also have been momentarily activated by the narrative experience, rather than as a construct completely unaffected by the film.

#### 2.2.3 Prosocial behavior measure

Developed by Gustavo Carlo and Brandy A. Randall in 2002, this scale is based on the multidimensional theory of prosocial tendencies, which posits that prosocial tendencies are not a single construct but encompasses six forms with different motivations and contexts. The scale includes six dimensions: public prosocial, anonymous prosocial, emergency prosocial, emotional prosocial, compliant prosocial, and altruism, with a total of 23 items. A 5-point Likert scale (1 = not at all like me, 5 = very much like me) is used for scoring. Average scores for each dimension and a total score can be calculated. Internal consistency: total scale Cronbach’s *α* = 0.81-0.92. Alpha coefficients for each dimension: public (0.72), anonymous (0.80), emergency (0.81), emotional (0.79), compliant (0.77), altruism (0.84). The prosocial behavior scale (used here to measure prosocial tendencies) has been widely used in cross-cultural research, and its Chinese version has demonstrated acceptable psychometric properties in prior studies with Chinese samples (33). However, cultural variations in the conceptualization of prosocial tendencies—particularly the greater emphasis on relational and collectivistic forms of helping in Chinese culture—suggest that future research may benefit from culturally adapted measures that capture these nuances (33, 34). The internal consistency coefficients obtained in this study: total scale Cronbach’s *α* = 0.84. Alpha coefficients for each dimension: public (0.92), anonymous (0.80), emergency (0.76), emotional (0.78), compliant (0.89), altruism (0.86). These values were broadly consistent with those reported in previous research.

### 2.3 Procedure

All participants completed the same procedure. No experimental manipulation or random assignment was involved. Data were collected in the institutional micro-teaching suite, a dedicated research space comprising 10 independent observation rooms, each with a capacity of approximately 30 participants. The researchers obtained duplicate keys from the facility management, thereby enabling access to the laboratory between 6:00 a.m. and 11:00 p.m. during the study period. Approximately 30 participants attended each session in a micro-classroom, all participants were uniformly instructed by the researchers as follows: “This aesthetic education session features a highly engaging film, *Dying to Survive*, which you are requested to view with full attention. To ensure both the quality of your viewing experience and the valid assessment of your aesthetic responsiveness, a questionnaire concerning the film content will be administered immediately after the screening, with an estimated completion time of approximately 30 minutes. The survey does not collect any personally identifiable information and poses no risk to your privacy. Accordingly, you are encouraged to provide answers based on your genuine first responses and personal preferences.” Then the film *Dying to Survive* was played on a large screen. After the film, participants completed the narrative transportation scale, the character identification questionnaire, the moral identity questionnaire, and the prosocial tendencies scale. The entire procedure was conducted during the course to maintain continuity.

### 2.4 Statistical analysis

All analyses were conducted in SPSS 27.0 and Hayes (27) PROCESS macro; confirmatory factor analyses were conducted in AMOS 26.0. Descriptive statistics (means, standard deviations) were computed for all study variables, and associations among narrative transportation, character identification, moral identity, and prosocial tendencies were assessed using Pearson correlation coefficients. Internal consistency was evaluated with Cronbach’s α, composite reliability (CR), and average variance extracted (AVE). Common method bias was examined using Harman’s single-factor test (unrotated principal component analysis; criterion: first factor < 40%; 35) and the unmeasured latent method construct (ULMC) approach. For the CFAs, items within each multidimensional scale were assigned to three parcels using the item-to-construct balancing technique, yielding three indicators per construct (36); discriminant validity was assessed with the Fornell–Larcker criterion and the HTMT ratio (37). The indirect association was tested with PROCESS Model 4, and the moderated mediation model with PROCESS Model 14, using 5,000 bootstrap resamples and 95% bias-corrected confidence intervals (27, 38). Character identification and moral identity were mean-centered prior to computing the interaction term (39), and moderation was evaluated via the significance of the interaction term (40). Significant interactions were probed with conditional effects at the 16th, 50th, and 84th percentiles of the moderator and Johnson–Neyman significance regions. Model diagnostics included variance inflation factors (threshold < 5; 41), the Breusch–Pagan test for heteroskedasticity, and skewness/kurtosis of residuals (|values| < 1). *Post hoc* power analyses were conducted with G*Power 3.1.9.7 following Fritz and MacKinnon (42) and Regorz (43). The significance level was set at α = .05 (two-tailed).

Given that G*Power 3.1.9.7 does not provide a direct function for estimating sample size for the index of moderated mediation, we followed the recommendations of Fritz and MacKinnon (42) and Regorz (43) by decomposing Model 14 into two sub-regression equations and conducting *post hoc* power analyses. For the second-stage moderation analysis, the incremental explanatory power of the interaction term M×W on the dependent variable Y was tested after controlling for the independent variable X, mediator M, and moderator W. The results indicated that with the current sample size of *N* = 1054, the statistical power was 0.996 when the effect size was *f*² = 0.02, and 0.900 when *f*² = 0.01. The minimum detectable effect size at a power of 0.80 was *f*² = 0.0075. For the a path (X → M), the power was greater than 0.999 at *f*² = 0.02. Collectively, the sample size was sufficient to detect small-to-moderate moderated mediation effects.

Observed-variable (composite-score) analyses were used for three reasons. First, all four measures are established instruments whose reliability was adequate in this sample (α = 0.70–0.85; CR = 0.84–0.87). Second, composite-score regression with bootstrapped indirect effects (PROCESS; 27) remains the prevailing analytic approach in narrative-persuasion research, and bootstrap confidence intervals do not assume normally distributed sampling distributions (38). Third, the parceled measurement model indicated adequate convergent validity, supporting the use of scale composites. Nevertheless, the marginal discriminant validity between narrative transportation and prosocial tendencies is acknowledged as a limitation, and latent-variable replication is recommended for future research.

### 2.5 Ethics statement

The study followed the Declaration of Helsinki. Formal ethical review was waived by the institutional review boards of Sichuan University of Media and Communications, Chengdu College of Arts and Sciences due to the anonymous survey design. All participants provided written informed consent.

## 3 Results

### 3.1 Descriptive statistics and correlation analysis

The means, standard deviations, and correlation coefficients for narrative transportation, character identification, moral identity, and prosocial tendencies are presented in **Table 1**. Narrative transportation, character identification, moral identity, and prosocial tendencies were all significantly and positively correlated.

**Table 1:** Descriptive statistical analysis table (*N* = 1054).

Variable	*M*	*SD*	1	2	3	4
1. Narrative transportation	3.99	0.71	1			
2.Character identification	3.17	0.51	0.22**	1		
3. Moral identity	3.34	0.53	0.08*	0.09**	1	
4. Prosocial tendencies	2.91	0.47	0.67**	0.23**	0.03	1

*p < 0.05, **p < 0.01. The four scales have different scoring ranges and different numbers of items, the mean and standard deviation were calculated using the total score divided by the number of items for each scale.

Correlational analyses revealed that narrative transportation was significantly correlated with prosocial tendencies (*r* = 0.67, *p* < 0.01), and character identification (*r* = 0.22, *p* < 0.01), and the mediator was significantly correlated with the dependent variable (*r* = 0.23, *p* < 0.01). These associations were consistent with the hypothesized mediation model. The non-significant correlation between the moderator “moral identity” and the dependent variable (*r* = 0.03, *p* > 0.05) does not preclude moderation analysis. Thus, further moderation analysis is statistically warranted.

### 3.2 Common method bias

The Harman single-factor test extracted 14 factors with eigenvalues greater than 1. The variance explained by the first unrotated common factor was 19.20%, which is below the critical standard of 40%.

The parceled four-factor model showed markedly improved fit: *χ²*(48) = 723.12, CFI = 0.909, TLI = 0.875, RMSEA = 0.116, SRMR = 0.073. Standardized loadings ranged from 0.70 to 0.92; composite reliability (CR) ranged from 0.84 to 0.87 and average variance extracted (AVE) from 0.64 to 0.69, exceeding conventional thresholds (44). We acknowledge that TLI and RMSEA did not fully meet conventional cutoffs (45), which we attribute in part to the large sample (*N* = 1,054) and the strong association between narrative transportation and prosocial tendencies (see below); these indices are therefore interpreted with caution.

A single-factor model fitted to the same 12 parcels fit substantially worse (CFI = 0.514, TLI = 0.406, RMSEA = 0.252, SRMR = 0.186), indicating that a single common factor cannot adequately account for the covariance among the indicators (46). We further re-estimated the unmeasured latent method construct (ULMC) model on the parceled indicators. Adding an orthogonal common method factor improved overall fit (CFI = 0.965 vs. 0.909; ΔCFI = 0.055) (46), indicating that some method variance is likely present; however, the improvement was concentrated in the narrative-transportation and prosocial-tendencies parcels, the augmented model yielded an improper solution (a negative standardized trait loading for one prosocial-tendencies parcel), and the character-identification and moral-identity indicators retained strong loadings on their substantive factors (0.74–0.91). Taken together with the very poor fit of the single-factor model reported above, these results suggest that common method bias alone does not account for the observed associations, although—as in any single-source, self-report design—it cannot be ruled out entirely.

The square roots of the AVEs (0.80–0.83) exceeded all inter-construct correlations except that between narrative transportation and prosocial tendencies. Consistently, HTMT values were below.45 for all construct pairs except narrative transportation–prosocial tendencies (HTMT = .86, marginally above the.85 criterion). This indicates that discriminant validity between these two constructs is only partially established—unsurprising, given that both were assessed immediately after viewing within the same affective context—and the strong total association between them (*r* = 0.67) should therefore be interpreted cautiously. Notably, the constructs central to this study’s novel contribution—the moderation of the character identification–prosocial tendencies association by moral identity—showed clear discriminant validity from all other constructs.

### 3.3 Mediation analysis (PROCESS macro model 4)

As shown in **Table 2**, narrative transportation was positively associated with character identification (path a: *b* = 0.156, *p* < 0.001), and character identification was positively associated with prosocial tendencies (path b: *b* = 0.076, *p* < 0.001). More critically, the bootstrap 95% confidence interval for the indirect effect (a × b) excluded zero (indirect effect = 0.012, Boot 95% CI [0.005, 0.020]), indicating a statistically significant indirect association of narrative transportation on prosocial tendencies through character identification. The total effect of narrative transportation on prosocial tendencies was 0.438 (*SE* = 0.0150, *p* < 0.001), and the direct effect was 0.426. The indirect effect accounted for 2.72% of the total effect. These results are consistent with the hypothesized indirect association of character identification, suggesting that character identification may be one pathway linking narrative transportation and prosocial tendencies.

**Table 2:** Mediation path coefficients (unstandardized).

Path	*Effect*	*SE*	*t*	*p*	LLCI	ULCI
Narrative transportation→Character identification(a)	0.156	0.021	7.345	< 0.001	0.115	0.198
Character identification → Prosocial tendencies (b)	0.076	0.021	3.540	< 0.001	0.034	0.119
Narrative transportation → Prosocial tendencies (c’)	0.426	0.015	27.929	< 0.001	0.396	0.456
Narrative transportation → Prosocial tendencies (c)	0.438	0.015	29.278	< 0.001	0.409	0.468

*SE*, standard error; LLCI and ULCI , lower and upper limits of the 95% bootstrap confidence interval (5,000 resamples).

Diagnostic tests indicated that the variance inflation factor (VIF) values for all predictor variables were below 2, well below the recommended threshold of 5 (41). The Breusch–Pagan test was not significant (χ² = 0.73, *p* = 0.695), suggesting that severe heteroskedasticity was not a concern. The absolute values of skewness and kurtosis of the residuals were both less than 1, satisfying the assumption of normality. Collectively, these findings indicate that the model estimates in this study are reasonably robust.

### 3.4 Moderated mediation analysis (PROCESS macro model 14)

Narrative transportation was significantly and positively associated with character identification (see **Table 3**, Model 2) (*b* = 0.156, *SE* = 0.021, *t*(1052) = 7.345, *p* <.001, 95% CI [0.115, 0.198]), suggesting that higher levels of narrative transportation were associated with greater character identification. This model accounted for 4.9% of the variance in character identification (*R²* = .049, *F*(1, 1052) = 53.949, *p* <.001).

**Table 3:** The moderating role of moral identity in the relationship between character identification and prosocial tendencies (examining a moderated mediation model).

Variable	Model 2 [character identification (centered)]				Model 3 (prosocial tendencies)			
	*Coefficient*	*SE*	*t*	95% CI	*Coefficient*	*SE*	*t*	95% CI
Narrative transportation	0.156***	0.021	7.345	[0.115, 0.198]	0.427***	0.015	27.996	[0.397, 0.457]
Character identification (Centered)					0.078***	0.022	3.607	[0.035, 0.120]
Moral identity (Centered)					-0.027	0.020	-1.348	[-0.067, 0.012]
Character identification (Centered) × Moral identity (Centered)					0.106***	0.040	2.637	[0.027, 0.186]
R²	0.049				0.460			
Adjusted R²	0.048				0.458			
F	F(1, 1052) = 53.949, p < 0.001				F(4, 1049) = 223.186, p < 0.001			

Bootstrap confidence intervals based on 5,000 resamples. X, Narrative Transportation; M, Character identification; Y, Prosocial tendencies.*p < 0.05, **p < 0.01, ***p < 0.001.

As presented in **Table 3** (Model 3), the direct association between narrative transportation and prosocial tendencies was significant (*b* = 0.427, *SE* = 0.015, *t*(1049) = 27.996, *p* < 0.001, 95% CI [0.397, 0.457]), indicating that narrative transportation continued to show a direct positive association with prosocial tendencies after controlling for the mediator and moderator. The main association between character identification and prosocial tendencies was significant (*b* = 0.078, *SE* = 0.022, *t*(1049) = 3.607, p <.001, 95% CI [0.035, 0.120]), indicating that character identification was positively associated with prosocial tendencies. The main association between moral identity and prosocial tendencies was not significant (*b* = −0.027, *SE* = 0.020, *t*(1049) = −1.348, *p* = 0.178, 95% CI [−0.067, 0.012]). Critically, the interaction term character identification × moral identity was significant (*b* = 0.106, *SE* = 0.040, *t*(1049) = 2.637, *p* = .009, 95% CI [0.027, 0.186]), indicating that moral identity significantly moderated the relationship between character identification and prosocial tendencies. The inclusion of this interaction term yielded a significant increase in *R²* of 0.0036 (*ΔR²* = .0036, *F*(1, 1049) = 6.956, *p* = 0.009).

The conditional associations between character identification and prosocial tendencies at the 16th, 50th, and 84th percentiles of moral identity are presented in **Table 4**. At low levels of moral identity, the association between character identification and prosocial tendencies was not significant (*b* = 0.013, *SE* = 0.033, *t* = 0.398, *p* = .691, 95% CI [−0.051, 0.077]). In contrast, at moderate and high levels of moral identity, the associations between character identification and prosocial tendencies were positive and significant (*b* = 0.090 and 0.139, respectively, *p* < 0.001), with the magnitude of the associations increasing as moral identity increased. Johnson–Neyman significance regions further revealed that the association between character identification and prosocial tendencies was significant when moral identity ≥ −0.2804 (mean-centered; approximately 0.53 *SD* below the mean), a range that comprised approximately 67.8% of the sample; when moral identity fell below this value, the association was not significant. Collectively, these findings suggest that the positive association between character identification and prosocial tendencies was weaker at low levels of moral identity, whereas higher levels of moral identity progressively strengthened the positive relationship between character identification and prosocial tendencies. This pattern is visualized in **Figure 2**.

**Table 4:** Conditional associations between character identification and prosocial tendencies at different levels of moral identity.

Moral identity Level	Description	*Effect*	*SE*	*t*	*p*	*95% CI*
−0.6078 (−1 *SD*)	16th percentile	0.013	0.033	0.398	0.691	[−0.051, 0.077]
0.1122 (Median)	50th percentile	0.090	0.022	4.078	< 0.001	[0.047, 0.133]
0.5722 (+1 *SD*)	84th percentile	0.139	0.032	4.403	< 0.001	[0.077, 0.200]

**Figure 2:**
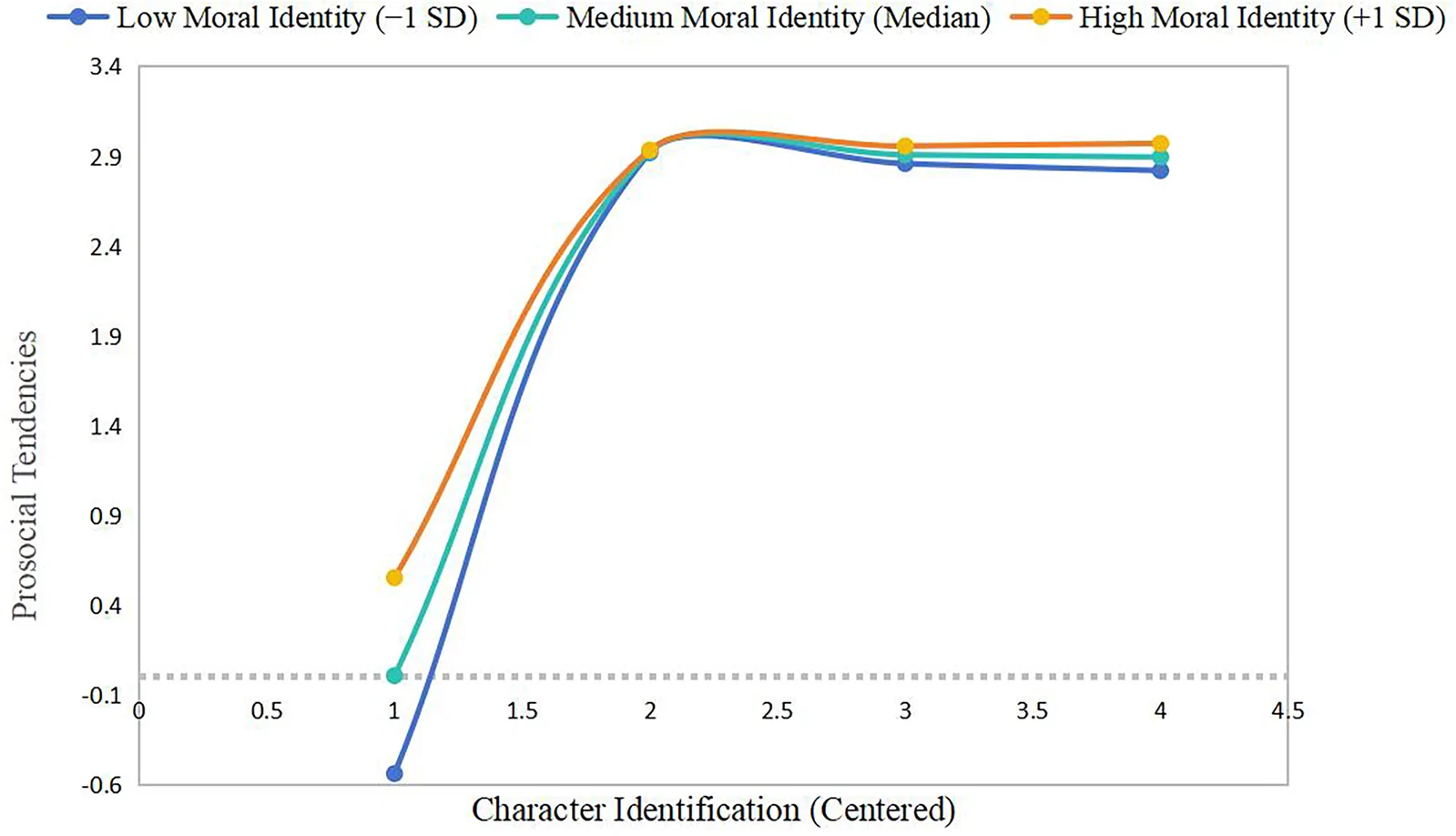
Simple slopes plot of the moderating role of moral identity on the relationship between character identification and prosocial tendencies.

The conditional indirect effects of narrative transportation on prosocial tendencies via character identification at different levels of moral identity were as follows. At low levels of moral identity, the indirect effect was not significant (*effect* = 0.002, Boot*SE* = 0.005, 95% BootCI [−0.008, 0.012]), as the confidence interval contained zero. At moderate levels of moral identity, the indirect effect was significant (*effect* = 0.014, Boot*SE* = 0.004, 95% BootCI [0.007, 0.023]), with the confidence interval excluding zero. At high levels of moral identity, the indirect effect was also significant (*effect* = 0.022, Boot*SE* = 0.006, 95% BootCI [0.011, 0.034]), with the confidence interval again excluding zero. The index of moderated mediation was 0.017 (Boot*SE* = 0.007, 95% BootCI [0.005, 0.031]), and the confidence interval did not include zero, providing further evidence for a significant moderated mediation pattern. In other words, moral identity significantly moderated the indirect path from narrative transportation to prosocial tendencies via character identification.

## 4 Discussion

Drawing upon narrative transportation theory and character identification theory, this study constructed a moderated mediation model to examine the association between cinematic narrative transportation and prosocial tendencies among college students, focusing on the indirect role of character identification and the moderating role of moral identity. The results indicated that narrative transportation was significantly and positively associated with prosocial tendencies, with character identification serving as a significant indirect pathway. Furthermore, the second stage of this indirect pathway was moderated by moral identity. Specifically, for individuals with higher moral identity, the positive association between character identification and prosocial tendencies was stronger, and the indirect effect of narrative transportation on prosocial tendencies through character identification was more pronounced; for those with lower moral identity, this indirect effect was relatively weaker. These findings suggest that narrative media may be associated with moral reflection and identify moral identity as a potential boundary condition for narrative persuasion processes. The indirect pathway through character identification accounted for only 2.72% of the total association. Although this proportion is modest, the bootstrap confidence interval excluded zero, indicating that the indirect pathway was statistically detectable. However, this statistical detectability should not be equated with substantive importance. Several considerations help contextualize this small effect size, although they do not change the fact that the effect was modest. First, prosocial tendencies are a multidimensional construct shaped by a constellation of dispositional, social, and situational factors (34). Within such a complex nomological network, it is not surprising that a single mediator—here, character identification elicited by a two-hour film—accounted for only a small portion of the variance. Nevertheless, the small magnitude of this indirect effect should be acknowledged. Second, the modest indirect effect may reflect the gap between narrative persuasion and actual behavioral enactment: while identifying with a morally growing character may be associated with viewers’ value orientations and prosocial tendencies, linking these psychological responses to actual prosocial behavior requires the confluence of multiple enabling conditions (e.g., social cues, opportunity structures, and habit strength). Third, from an educational and applied perspective, small indirect effects might accumulate across repeated exposures or over time. However, this possibility is speculative and cannot be directly tested in the present single-exposure, cross-sectional design. For instance, a 2.72% indirect pathway, if operating consistently across multiple film-based aesthetic education sessions, might accumulate into more noticeable changes in students’ prosocial orientation. However, this remains a hypothesis for future longitudinal research. Thus, rather than dismissing the small effect size, we interpret it as a statistically detectable but modest indirect pathway that warrants further investigation.

First, although previous research has separately examined narrative transportation, character identification, and prosocial tendencies, few studies have integrated them into a single model to test the mediating role of character identification (16, 18). Using a large sample of college students (*N* = 1054), the present study tested this indirect pathway, providing empirical evidence consistent with narrative persuasion theory. Unlike prior laboratory-based studies using brief narratives (18, 31), this study used a full-length commercial feature film viewed in a classroom setting, which more closely approximates real-world viewing conditions; nonetheless, the use of a single film limits the generalizability of the findings.

Second, this study is among the first to examine the moderating role of moral identity in the link between character identification and prosocial tendencies within a cinematic context, moral identity—as a core component of self-concept—may function as a boundary condition that moderates whether individuals link character identification with prosocial motivation. Whereas prior research has largely focused on moral identity in traditional moral judgment and decision-making (22), the present study extends its application into the interdisciplinary domain of aesthetic experience and media reception.

Third, at the applied level, this study was situated within an authentic educational context of film-based aesthetic education rather than relying on laboratory-manipulated stimuli. The results suggest that selecting films featuring moral dilemmas and character growth arcs (e.g., *Dying to Survive*) in college aesthetic curricula may be associated with students’ moral reflection and prosocial motivation, offering a preliminary, statistically grounded account of a psychological process potentially involved in the educational function of cinema. In contrast to prior work emphasizing cognitive pathways of narrative persuasion (47), this study highlights the interplay between emotional involvement (Character identification) and individual differences (moral identity), enriching our understanding of how narratives relate to audience responses.

Narrative transportation theory posits that high immersion in a narrative reduces critical scrutiny, making individuals more receptive to embedded moral messages (18). The present findings are consistent with this proposition: viewers with higher narrative transportation reported significantly greater identification with the protagonist, Cheng Yong. His transformation from a profit-oriented merchant to a “medicine hero” in *Dying to Survive* constitutes a moral growth arc that may have been associated with emotional resonance and moral identity, corresponding to stronger viewers’ psychological connection to the character.

Regarding the mediating role of character identification, the results align with the empathy-altruism hypothesis (21) and the self-expansion model (25, 26). Character identification blurs the psychological boundary between the self and the character, incorporating the character’s goals and behaviors into the self-concept. When viewers identify with an altruistic character, they may internalize the character’s moral choices as part of their own behavioral tendencies, which may be associated with stronger intentions. Although the indirect effect accounted for only 2.72% of the total association, it was statistically robust (Bootstrap 95% CI excluding zero). This relatively modest effect size may reflect that prosocial tendencies are a multidimensional construct (34) shaped by multiple factors, with narrative experience being one of many contributing factors. Nevertheless, the indirect association remained significant, suggesting that character identification is a statistically detectable—though not exclusive—pathway linking narrative transportation to prosocial tendencies.

Regarding the moderating role of moral identity, the findings are consistent with self-consistency theory (23, 24). Individuals with higher moral identity, for whom moral traits are more central to self-concept, are more likely to show intrinsic motivation to maintain self-concept consistency after incorporating a character’s altruistic actions, which may correspond to stronger prosocial tendencies. Conversely, for individuals with lower moral identity, even if they identify with a character, the lack of moral trait salience may be associated with weaker behavioral motivation—indeed, when moral identity was low (−1 SD), the association between character identification and prosocial tendencies was non-significant (b = 0.013, p = 0.691).Johnson–Neyman analysis further revealed that character identification was significantly associated with prosocial tendencies only when mean-centered moral identity ≥ −0.28 (approximately 67.8% of the sample). This pattern indicates that the association between character identification and prosocial tendencies was not statistically significant in the lower range of moral identity. We note, however, that this value is a region of statistical significance identified by the Johnson–Neyman procedure rather than a substantively meaningful psychological threshold, and it should not be overinterpreted. Descriptively, this pattern offers a nuanced perspective on how individual differences moderate narrative persuasion processes. An important caveat concerns the temporal ordering of moral identity. Although moral identity is theoretically regarded as a dispositional construct, it was measured after the film-viewing session. It is therefore possible that the film temporarily increased the accessibility of moral identity, rather than that moral identity functioned as a purely stable moderator. This possibility does not invalidate the observed moderating pattern, but it does suggest that the present findings should be interpreted with caution. Future research using pretest–posttest or experimental designs is needed to disentangle stable individual differences in moral identity from transient narrative-induced activation.

A related issue concerns the magnitude of the interaction effect: the addition of the character identification × moral identity term explained only an incremental 0.36% of the variance in prosocial tendencies (*ΔR²* = 0.0036). While this increment is small in absolute terms, its interpretation should be grounded in the nature of interaction effects in psychological research. As noted by McClelland and Judd (48) and Aguinis and Stone-Romero (49), interaction effects in field studies with non-manipulated continuous variables are inherently difficult to detect, and even small *R²* increments can denote theoretically meaningful moderation patterns. In the present context, the small *ΔR²* should not be overinterpreted, but it does suggest that the moderating role of moral identity is theoretically meaningful: it reveals that the association between character identification and prosocial tendencies is not universal but contingent upon viewers’ moral self-concept. The Johnson–Neyman analysis further showed that the identification–tendencies association was not statistically significant in the lower range of moral identity (below −0.28 mean-centered, approximately 32% of the sample). Descriptively, this pattern suggests that the association between film-based aesthetic education and prosocial tendencies was weaker among students with lower moral identity; whether additional scaffolding (e.g., pre-viewing moral reflection or post-viewing reflective discussions) benefits these students remains a speculative possibility that requires experimental testing. Future research should therefore explore other potential moderators—such as empathy, perspective-taking, or narrative engagement style—that might amplify the character identification–prosocial tendencies linkage and yield larger interaction effects.

## 5 Research limitations

This study has certain limitations. Firstly, the participants were university students, and the sample source was relatively homogeneous. Future research can be further expanded to groups of different ages and cultural backgrounds to test the universality of the research results. Secondly, this study measured prosocial tendencies using a self-report scale (34), which captures participants’ perceived prosocial tendencies rather than their actual behavioral enactment in real-world settings. This distinction is important for several reasons. Theoretically, our model was designed to explain the psychological process through which narrative immersion is associated with behavioral intention and self-perceived tendency, which is a necessary precursor to actual behavior. Thus, the self-report measure aligns with the immediate aftermath of film viewing, where intention rather than long-term behavioral observation is the appropriate outcome. However, we acknowledge that self-reported prosocial tendencies may be subject to social desirability bias and may not perfectly correspond to actual helping actions in daily life. In this regard, our findings should be interpreted as evidence of an association between narrative exposure and motivational changes, rather than as evidence of directly observed behavioral outcomes. Future research would benefit from complementing self-report measures with peer nominations, experience sampling, or behavioral tasks (e.g., donating money or time) to capture the full spectrum of prosocial tendencies and to test the temporal persistence of narrative-related associations. In addition, although the indirect effect was statistically significant, its effect size was small (2.72% of the total association). This finding should therefore be interpreted in terms of statistical detectability rather than substantive magnitude. Moreover, although the parceled measurement model showed acceptable fit and adequate convergent validity, discriminant validity between narrative transportation and prosocial tendencies was only partially established (HTMT = .86), and the TLI and RMSEA values of the measurement model did not fully meet conventional cutoffs. These measurement issues should be taken into account when interpreting the findings. Future research should replicate the model using latent-variable approaches and measures with stronger discriminant evidence. Third, this study only selected *Dying to Survive* as the viewing material. Although this film has typical characteristics of moral conflict and character transformation, the associations of different narrative types and character structures with audience responses may vary. Future research can further compare the mechanisms of action of various types of films. Fourth, moral identity was measured after the film-viewing session. Although it is theoretically conceptualized as a relatively stable construct, the present design cannot rule out the possibility that the film temporarily activated moral identity. Future research should include a pre-film measure of moral identity or use experimental designs to examine whether narrative exposure changes moral identity itself. Finally, this study used a cross-sectional design, which does not permit causal inference. Future research can use longitudinal designs or experimental methods to examine the causal relationships implied by the model.

## 6 Conclusions

This study examined a hypothesized moderated mediation model linking cinematic narrative transportation to prosocial tendencies among Chinese college students. Three principal findings emerged. First, narrative transportation was positively associated with prosocial tendencies, both directly and indirectly. Second, character identification showed a significant indirect association linking narrative transportation with prosocial tendencies, consistent with a partial mediation pattern: psychologically “becoming” the character is one pathway through which narrative immersion is connected to real-world prosocial tendencies. Third, moral identity moderated the second stage of this pathway: the association between character identification and prosocial tendencies, and the corresponding indirect association, were significant only among students with moderate-to-high levels of moral identity, consistent with the characterization of moral identity as a moderating factor in the transportation–identification–behavior pattern, while acknowledging that moral identity was assessed after viewing and thus may partly reflect narrative-induced activation.

Theoretically, these findings bring together narrative transportation theory, character identification theory, and self-consistency theory in a single model of narrative persuasion, and they extend this research tradition to a full-length commercial feature film viewed in an authentic aesthetic-education context, although the single-film, single-context design calls for replication with diverse materials. The results also highlight the value of distinguishing a situationally inducible mechanism - character identification - from a dispositional boundary condition such as moral identity, thereby specifying not only how but also for whom narrative experience is associated with prosocial motivation.

Practically, although any applied implications must remain tentative given the observational, single-film, and self-report nature of the study, the findings are consistent with a stratified educational logic. Because the indirect association was concentrated among students with moderate-to-high moral identity, film selection alone may not suffice: pairing films featuring moral dilemmas and character growth arcs (e.g., Dying to Survive) with moral-identity cultivation and guided post-viewing reflection may be more strongly associated with prosocial intentions than screening alone. At the institutional level, aesthetic-education curricula that combine immersive viewing with structured discussion may help students link emotional identification with deliberate prosocial intentions. These possibilities are hypothesis-generating rather than prescriptive and await experimental and longitudinal evaluation.

Several directions for future research follow from this work. Longitudinal and experimental designs are needed to establish the causal ordering implied by the hypothesized model, and behavioral tasks or third-party reports of prosocial tendencies would complement self-report measures. Future studies should compare diverse narrative types and character structures, and replicate the model across different age groups and cultural contexts. Given the central role of film in college aesthetic education, understanding the psychological conditions under which narrative immersion is associated with prosocial tendencies has direct educational relevance. We hope the present findings inform both the research agenda on narrative persuasion and the design of film-based moral and aesthetic education programs.

## Data Availability

The original contributions presented in the study are included in the article/**Supplementary Material**. Further inquiries can be directed to the corresponding author.

## References

[1] Bordwell D . Narration in the Fiction Film. Madison, Wis.: University of Wisconsin Press (1985).

[2] Chatman S . Story and Discourse: Narrative Structure in Fiction and Film. Ithaca, N.Y.: Cornell University Press (1978).

[3] Brewer WF Lichtenstein E . Stories are to entertain: A structural-affect theory of stories. J Pragmatics. (1982) 6:473–86. doi: 10.1016/0378-2166(82)90021-2

[4] Lang A . The limited capacity model of mediated message processing. J Communication. (2000) 50:46–70. doi: 10.1111/j.1460-2466.2000.tb02833.x40046247

[5] Nabi RL Green C . The role of a narrative's emotional flow in promoting persuasive outcomes. Media Psychol. (2015) 18:137–62. doi: 10.1080/15213269.2014.912585

[6] Oliver MB Bartsch A . Appreciation as audience response: Exploring entertainment gratifications beyond hedonism. Hum Communication Res. (2010) 36:53–81. doi: 10.1111/j.1468-2958.2009.01368.x40046247

[7] Loewenstein G . The psychology of curiosity: A review and reinterpretation. psychol Bull. (1994) 116:75–98. doi: 10.1037/0033-2909.116.1.7527371692

[8] China Film Administration . 2024 annual film box office statistics (2025). Available online at: https://www.chinafilm.gov.cn/xwzx/gzdt/202501/t20250102_879736.html (Accessed October 7, 2026).

[9] Institute of Psychology, Chinese Academy of Sciences . Report on National Mental Health Development of China (2021–2022). In: Mental Health Blue Book. Beijing: Social Sciences Academic Press (2023).

[10] Eisenberg N Mussen PH . The Roots of Prosocial Behavior in Children. Cambridge [England]; New York: Cambridge University Press (1989).

[11] Penner LA Dovidio JF Piliavin JA Schroeder DA . Prosocial behavior: Multilevel perspectives. Annu Rev Psychol. (2005) 56:365–92. doi: 10.1146/annurev.psych.56.091103.070141 15709940

[12] Martínez MÁ . Empathy versus engagement: A storyworld possible selves approach to narrative discourse. In: Gómez González MÁ Mackenzie JL , editors. The Construction of Discourse as Verbal Interaction. Amsterdam; Philadelphia: John Benjamins Publishing Company (2018). p. 251–72.

[13] Lankhuizen T . From Frames to Feelings: Visual Form, Empathy, and Narrative Engagement in Hollywood Cinema [Doctoral dissertation]. Amsterdam: Vrije Universiteit Amsterdam (2025).

[14] Chen T Lin JS . Entertainment-education of altruistic behaviors: An empirical study of the effects of the narrative persuasion of a nature conservation film. Chin J Communication. (2014) 7:373–88. doi: 10.1080/17544750.2014.94643037339054

[15] Ballard C . (1996). Fly Away Home. Columbia Pictures.

[16] Murphy ST Frank LB Moran MB Patnoe-Woodley P . Involved, transported, or emotional? Exploring the determinants of change in knowledge, attitudes, and behavior in entertainment-education. J Communication. (2011) 61:407–31. doi: 10.1111/j.1460-2466.2011.01554.x40046247

[17] Zhao D Raney AA . Exploring the effects of positive emotions and attribution on helping behaviors after exposure to meaningful and pleasurable movies. Communication Res Rep. (2023) 40:261–71. doi: 10.1080/08824096.2023.226908537339054

[18] Green MC Brock TC . The role of transportation in the persuasiveness of public narratives. J Pers Soc Psychol. (2000) 79:701–21. doi: 10.1037/0022-3514.79.5.701 11079236

[19] Green MC Brock TC . In the mind's eye: Transportation-imagery model of narrative persuasion. In: Narrative Impact. Mahwah, NJ: Psychology Press (2000). p. 315–41.

[20] Cohen J . Defining identification: A theoretical look at the identification of audiences with media characters. In: Advances in Foundational Mass Communication Theories. Abingdon, Oxon: Routledge (2018). p. 253–72.

[21] Batson CD . The Altruism Question: Toward a Social-Psychological Answer. Hillsdale, NJ: Psychology Press (2014).

[22] Aquino K Reed A II . The self-importance of moral identity. J Pers Soc Psychol. (2002) 83:1423–40. doi: 10.1037/0022-3514.83.6.1423 12500822

[23] Lecky P . Self-Consistency: A Theory of Personality. New York, N.Y.: Island Press (1945).

[24] Aronson E . The theory of cognitive dissonance: A current perspective. In: Berkowitz L , editor. Advances in Experimental Social Psychology, vol. 4. New York: Academic Press (1969). p. 1–34.

[25] Aron A Aron EN Tudor M Nelson G . Close relationships as including other in the self. J Pers Soc Psychol. (1991) 60:241–53. doi: 10.1037/0022-3514.60.2.24127371692

[26] Cialdini RB Brown SL Lewis BP Luce C Neuberg SL . Reinterpreting the empathy–altruism relationship: When one into one equals oneness. J Pers Soc Psychol. (1997) 73:481–94. doi: 10.1037/0022-3514.73.3.481 9294898

[27] Hayes AF . Introduction to Mediation, Moderation, and Conditional Process Analysis: A Regression-Based Approach. 3rd ed. New York, NY: Guilford Press (2022).

[28] Busselle R Bilandzic H . Measuring narrative engagement. Media Psychol. (2009) 12:321–47. doi: 10.1080/1521326090328725937339054

[29] Tang T Mahoney LM . Narrative engagement scale. In: Routledge eBooks. London: Routledge (2019). p. 309–14.

[30] Igartua JJ . Identification with characters and narrative persuasion through fictional feature films. Communications. (2010) 35:347–73. doi: 10.1515/comm.2010.01931755547

[31] Igartua JJ Barrios I . Changing real-world beliefs with controversial movies: Processes and mechanisms of narrative persuasion. J Communication. (2012) 62:514–31. doi: 10.1111/j.1460-2466.2012.01640.x40046247

[32] Ding X Zhang Z Bai J Guo B Jiang J Qiu Z . Translation, reliability, and validity of the Chinese version of the Moral Identity Scale in a sample of male offenders. Ethics Behav. (2025) 35:309–19. doi: 10.1080/10508422.2024.2377165

[33] Xiong W Lu Y Wang D Liu X Yao X He J . The development, validity and reliability of the Willingness of Prosocial Behavior Scale for community-dwelling older adults using Classical Testing Theory. Clin Interventions Aging. (2026) 21:576494. doi: 10.2147/cia.s576494 PMC12949800 41773184

[34] Carlo G Randall BA . The development of a measure of prosocial behaviors for late adolescents. J Youth Adolescence. (2002) 31:31–44. doi: 10.1023/a:101403303244041886696

[35] Podsakoff PM MacKenzie SB Lee J-Y Podsakoff NP . Common method biases in behavioral research: A critical review of the literature and recommended remedies. J Appl Psychol. (2003) 88:879–903. doi: 10.1037/0021-9010.88.5.879 14516251

[36] Little TD Cunningham WA Shahar G Widaman KF . To parcel or not to parcel: Exploring the question, weighing the merits. Struct Equation Modeling. (2002) 9:151–73. doi: 10.1207/s15328007sem0902_142819732

[37] Henseler J Ringle CM Sarstedt M . A new criterion for assessing discriminant validity in variance-based structural equation modeling. J Acad Marketing Sci. (2015) 43:115–35. doi: 10.1007/s11747-014-0403-8

[38] MacKinnon DP Lockwood CM Williams J . Confidence limits for the indirect effect: Distribution of the product and resampling methods. Multivar Behav Res. (2004) 39:99–128. doi: 10.1207/s15327906mbr3901_4 20157642 PMC2821115

[39] Wen ZL Hau KT Chang L . A comparison and application of moderating and mediating effects. Acta Psychologica Sin. (2005) 37:268–74. Available online at: https://journal.psych.ac.cn/xlxb/CN/Y2005/V37/I02/268 (Accessed October 7, 2026).

[40] Aiken LS West SG . Multiple Regression: Testing and Interpreting Interactions. Newbury Park, CA: Sage Publications (1991).

[41] Hair JF Ringle CM Sarstedt M . PLS-SEM: Indeed a silver bullet. J Marketing Theory Pract. (2011) 19:139–52. doi: 10.2753/mtp1069-6679190202

[42] Fritz MS MacKinnon DP . Required sample size to detect the mediated effect. Psychol Sci. (2007) 18:233–9. doi: 10.1111/j.1467-9280.2007.01882.x PMC2843527 17444920

[43] Regorz A . Sample size for a moderated mediation: Using G*Power. In: Regorz Statistik. Bochum, Germany: Regorz Statistik (2024). Available online at: https://www.regorz-statistik.de/en/power_moderated_mediation.html (Accessed October 7, 2026).

[44] Fornell C Larcker DF . Evaluating structural equation models with unobservable variables and measurement error. J Marketing Res. (1981) 18:39–50. doi: 10.1177/002224378101800104

[45] Wen Z Hau KT Marsh HW . Structural equation model testing: Cutoff criteria for goodness of fit indices and chi-square test. Acta Psychologica Sin. (2004) 36:186–94. Available online at: https://journal.psych.ac.cn/xlxb/CN/Y2004/V36/I02/186 (Accessed October 7, 2026).

[46] Cheung GW Rensvold RB . Evaluating goodness-of-fit indexes for testing measurement invariance. Struct Equation Modeling. (2002) 9:233–55. doi: 10.1207/s15328007sem0902_542819731

[47] Slater MD Rouner D . Entertainment-education and elaboration likelihood: Understanding the processing of narrative persuasion. Commun Theory. (2002) 12:173–91. doi: 10.1093/ct/12.2.173

[48] McClelland GH Judd CM . Statistical difficulties of detecting interactions and moderator effects. psychol Bull. (1993) 114:376–90. doi: 10.1037/0033-2909.114.2.376 8416037

[49] Aguinis H Stone-Romero EF . Methodological artifacts in moderated multiple regression and their effects on statistical power. J Appl Psychol. (1997) 82:192–206. doi: 10.1037/0021-9010.82.1.19227371692

